# Enhancing diagnostic accuracy in symptom-based health checkers: a comprehensive machine learning approach with clinical vignettes and benchmarking

**DOI:** 10.3389/frai.2024.1397388

**Published:** 2024-10-01

**Authors:** Leila Aissaoui Ferhi, Manel Ben Amar, Fethi Choubani, Ridha Bouallegue

**Affiliations:** ^1^Virtual University of Tunis, Tunis, Tunisia; ^2^Innov’Com Laboratory at SUPCOM, University of Carthage, Carthage, Tunisia; ^3^Faculty of Dental Medicine of Monastir, University of Monastir, Monastir, Tunisia

**Keywords:** health checker, symptoms, machine learning, confusion matrix, ROC/AUC curves, precision-recall curve, clinical vignettes, benchmarking

## Abstract

**Introduction:**

The development of machine learning models for symptom-based health checkers is a rapidly evolving area with significant implications for healthcare. Accurate and efficient diagnostic tools can enhance patient outcomes and optimize healthcare resources. This study focuses on evaluating and optimizing machine learning models using a dataset of 10 diseases and 9,572 samples.

**Methods:**

The dataset was divided into training and testing sets to facilitate model training and evaluation. The following models were selected and optimized: Decision Tree, Random Forest, Naive Bayes, Logistic Regression and K-Nearest Neighbors. Evaluation metrics included accuracy, F1 scores, and 10-fold cross-validation. ROC-AUC and precision-recall curves were also utilized to assess model performance, particularly in scenarios with imbalanced datasets. Clinical vignettes were employed to gauge the real-world applicability of the models.

**Results:**

The performance of the models was evaluated using accuracy, F1 scores, and 10-fold cross-validation. The use of ROC-AUC curves revealed that model performance improved with increasing complexity. Precision-recall curves were particularly useful in evaluating model sensitivity in imbalanced dataset scenarios. Clinical vignettes demonstrated the robustness of the models in providing accurate diagnoses.

**Discussion:**

The study underscores the importance of comprehensive model evaluation techniques. The use of clinical vignette testing and analysis of ROC-AUC and precision-recall curves are crucial in ensuring the reliability and sensitivity of symptom-based health checkers. These techniques provide a more nuanced understanding of model performance and highlight areas for further improvement.

**Conclusion:**

This study highlights the significance of employing diverse evaluation metrics and methods to ensure the robustness and accuracy of machine learning models in symptom-based health checkers. The integration of clinical vignettes and the analysis of ROC-AUC and precision-recall curves are essential steps in developing reliable and sensitive diagnostic tools.

## 1 Introduction

Symptom-based health checkers are a fascinating intersection of technology and healthcare offering accessible preliminary assessments based on reported symptoms. The concept operates in a straightforward manner: Symptoms are entered into the system, which then employs artificial intelligence (AI) algorithms such as machine learning (ML) to offer potential diagnoses or health recommendations. These tools have a rich history evolving from basic rule-based systems to sophisticated AI-driven models (Thani and Kasbe, 2022; Vida, 2022; Kumar and Meenakshi Sundaram, 2023; Wen and Huang, 2023). Historically, early health checkers were quite basic relying on predefined rules and simple decision trees. These systems were limited in scope and accuracy and often failing to account for the complexity and variability of human health conditions. However, the capabilities of these tools rose following the evolution of computational power and data availability (Beam and Kohane, 2018; Balogh and Adamkó, 2023).

The introduction of electronic health records (EHRs) and the explosion of medical data provided a fertile ground for more advanced models (Johnson et al., 2023). In recent years, the accuracy and reliability of health assessments have been significantly enhanced by health checkers utilizing ML and deep learning technologies (Tripathi and Goel, 2021). Modern symptom-based health checkers now incorporate various types of data, including user-reported symptoms, EHRs, and even data from wearable devices, making them more robust and comprehensive (Wynants et al., 2020). One notable example is the COVID-19 pandemic accelerating the development and adoption of symptom-based health checkers and many institutions developed AI-driven tools to help triage patients and manage healthcare resources more effectively (Hashemi et al., 2024). These tools demonstrated the potential of ML in rapidly evolving healthcare scenarios (Pogoncheff et al., 2023). There are many models and techniques used in these advanced health checkers. Decision trees and random forests remain popular for their interpretability and robustness across different datasets (Amorim et al., 2021). Enhanced with feature selection techniques and natural language processing (NLP) for handling free-text symptoms, these models have improved in understanding the complexity of user inputs (Jia et al., 2023). Deep learning models, especially convolutional neural networks (CNNs) and recurrent neural networks (RNNs), have taken center stage for their ability to capture intricate patterns in large datasets. CNNs are particularly effective for image-based diagnostics, while RNNs excel in handling sequential data, such as time-series health records (Shah et al., 2020).

The validation of these models is crucial. Cross-validation, especially k-fold cross-validation, remains a standard practice to ensure that the models generalize well to unseen data. This technique splits the data into k parts, trains the model k times, each time using a different part as the test set and the remaining parts as the training set, thus providing a comprehensive evaluation of the model’s performance (Shah et al., 2020; Chiu et al., 2024). Hyperparameter tuning is another critical aspect. Methods like grid search, random search, and bayesian optimization are employed to find the optimal parameters that enhance model performance and achieve the best possible predictive accuracy (Mavridou and Laws, 2004; Chicco and Jurman, 2020). To measure the effectiveness of these models, metrics like ROC (Receiver Operating Characteristic) curves and AUC (Area Under the Curve) are extensively used. These metrics are particularly useful in handling either imbalanced and balanced datasets, which are common in medical diagnostics. A high AUC value indicates that the model has a good ability to distinguish between different classes, such as healthy versus diseased (Chen et al., 2023; Liu et al., 2023). Ensemble methods have also shown great promise in recent years. Techniques like stacking, boosting, and bagging combine multiple models to improve predictive performance (Aissaoui Ferhi et al., 2019; Fei and Wang, 2020). Studies have demonstrated that ensemble methods can significantly enhance the accuracy and reliability of symptom-based health checkers compared to individual models (Veloski et al., 2005; García-Carretero et al., 2020). Explainable AI (XAI) is becoming increasingly important in healthcare. While complex models like deep learning offer high accuracy, they often operate as black boxes. XAI techniques aim to make these models more transparent, providing healthcare professionals with insights into how decisions are made. This transparency is crucial for clinical acceptance and trust in AI-driven diagnostics (You et al., 2022; Ahmad et al., 2023; Jia et al., 2023; Miao et al., 2024).

The integration of EHRs and patient-generated data has further advanced the capabilities of health checkers. By incorporating comprehensive datasets, these models can consider a wider range of patient histories and variables, leading to more accurate and personalized health assessments (Wynants et al., 2020; Griner et al., 2021; Johnson et al., 2023). Natural language processing (NLP) advancements have significantly improved the ability of health checkers to process and understand free-text inputs from users. Enhanced NLP algorithms can extract relevant information from unstructured data, which is a common way people report symptoms, thereby improving the performance and usability of these tools (Pogoncheff et al., 2023; Hashemi et al., 2024). Transfer learning, where models pre-trained on large datasets are fine-tuned on specific medical datasets, has also been a breakthrough. This approach gives models a head start, especially beneficial when dealing with limited medical data. It’s like leveraging prior knowledge to quickly adapt to new tasks (Amorim et al., 2021; Jia et al., 2023). The trend toward real-time data and continuous monitoring is also noteworthy. Wearable devices and mobile health apps generate a constant stream of health data that can be fed into these models for real-time monitoring and prediction. This capability not only enhances accuracy but also allows for timely interventions, potentially preventing diseases from progressing (Son et al., 2020; Alwazzan, 2023). Overall, symptom-based health checkers powered by ML represent a significant advancement in digital health. By leveraging sophisticated ML techniques, robust validation methods, and comprehensive data integration, these tools are becoming more reliable and accurate.

In the context of recent advancements in health checkers, our work stands out for its meticulous approach to enhance diagnostic accuracy and reliability. Our primary objectives have been to enhance the precision and robustness of symptom-based health checkers by leveraging high-quality datasets and validating performance using clinical vignettes. We have implemented rigorous validation techniques and analyzed performance using many metrics to ensure the models’ generalizability and reliability. We’ve taken our health checker to the next level by leveraging a high-quality dataset sourced from trusted medical repositories and methodically curated by a co-author who brings invaluable expertise as a practicing doctor making the dataset the backbone of our system and ensuring that our models are trained on robust and representative data. Additionally, we have tested the health checker using clinical vignettes, which are crucial for validation. Clinical vignettes (Veloski et al., 2005; García-Carretero et al., 2020) allow us to simulate real-world scenarios and evaluate the performance and accuracy of our health checker in a controlled, yet realistic setting. This comprehensive approach helps us fine-tune our models and ensures that our tool is reliable and effective in diverse clinical situations. We’ve also put our models through the wringer with rigorous validation techniques. We have subjected our models to an exhaustive 10-fold cross-validation to ensure their capacity to generalize across a diverse array of datasets and to overcome overfitting. Furthermore, we have undertaken a comprehensive optimization of our models’ hyperparameters to achieve best performance. Importantly, we have also engaged in a deep analysis of our models’ Area Under the Receiver Operating Characteristics (AUROC) and Area Under the Precision-Recall (AUPR) curves and scores which are rigorous diagnostic tools to quantify our models’ performance characteristics and to equip us with the insights necessary to fine-tune and validate their reliability. Our goal is to deliver a health checker that healthcare professionals and patients alike can rely on with confidence.

## 2 Methods

### 2.1 Data collection and domain understanding

The effectiveness of symptom-based health checkers is profoundly influenced by the quality and comprehensiveness of the data they utilize. Ensuring meticulous data collection from a multitude of trustworthy sources is vital as it enables the creation of representative and comprehensive datasets. Among the most valuable sources of data are EHRs due to their detailed clinical information, wearable devices due to the real-time health monitoring, and patient-reported outcomes due to nuanced understanding of health conditions (Wongvibulsin et al., 2019; Alzubaidi et al., 2023). In addition, public health databases maintained by organizations such as the CDC and WHO provide aggregated data on disease prevalence, health behaviors, and population health trends. Examples include the CDC’s Behavioral Risk Factor Surveillance System (BRFSS) and the WHO’s Global Health Observatory (GHO). These databases are mandatory for understanding broader health patterns and training symptom-based health checkers to recognize and predict public health issues effectively (Salvador et al., 2020; Mulchandani et al., 2022). Also, specialized health databases such as the UK Biobank and the National Health and Nutrition Examination Survey (NHANES) provide rich datasets that can be leveraged for health checker tools. The UK Biobank, for example, contains detailed health and genetic information from half a million UK participants, offering a comprehensive resource for studying the interplay between genetics, lifestyle, and health outcomes (Mavridou and Laws, 2004; Douaud et al., 2022). Some studies such as (Mavridou and Laws, 2004) identify and maps the expanding data collection strategies used in qualitative researches in the healthcare. In addition to collecting high-quality data, a deep understanding of the medical domain is equally important. Symptom-based health checkers need to be designed with a deep understanding of medical terminology, clinical workflows, and patient behavior patterns to ensure accurate data interpretation and clinically relevant recommendations (Machen, 2023; Aissaoui Ferhi et al., 2024). Analyzing patient behavior patterns is another critical aspect of domain understanding. This involves understanding how patients adhere to treatment plans, the frequency of their medical visits, and their lifestyle choices. Data from mobile apps and wearables can offer insights into these behaviors, enabling health checkers to deliver more personalized and effective health advice (Woodcock et al., 2021; Ozonze et al., 2023). The importance of clinical vignettes and clinical case reports in the validation process of symptom-based health checkers cannot be overstated. Clinical vignettes are detailed, hypothetical patient scenarios used to simulate real-life clinical encounters. Clinical case reports are detailed (Veloski et al., 2005) real patient scenarios and are used to describe and interpret the experienced symptoms and signs, final diagnosis, adapted treatment and follow up. In our article, we used the term “clinical vignette” to indicate either a clinical vignette or a clinical case report. By testing health checkers with these vignettes and case reports, researchers can evaluate the accuracy and reliability of the tool in a controlled realistic environment. This method helps identify potential gaps in the tool’s diagnostic capabilities and ensures it performs well across a variety of clinical scenarios, which is crucial for gaining clinical acceptance and trust. Our team is quite diverse including senior physician and senior researchers in information and communication technologies. The doctor’s medical expertise played a crucial role in ensuring the accuracy of the medical data we gathered. To really understand the medical diagnostic process, we delved into a range of resources, from medical books, public datasets,^1^^,^^2^ to trusted websites like ViDAL and Mayo Clinic.^3^^,^^4^ We also had some enlightening conversations with other medical professionals to grasp the real-world steps involved in diagnosing medical issues in general practice. Patient backgrounds, like age, gender, and medical history, were carefully incorporated into our dataset. To verify the robustness of our health checker, we tested a symptom dataset and numerous clinical vignettes (Marcio, 2024a; Marcio, 2024b) to enhance the comprehensiveness and applicability of the health checker.

### 2.2 Data preprocessing and feature engineering

Data preprocessing (Kale and Pandey, 2024) and feature engineering (GADA et al., 2021) are crucial steps in the development of a symptom-based health checker using ML. The aim is to prepare the collected dataset by cleaning it and converting raw data into a useful format for model training. Data cleaning involves handling missing values, removing duplicates, and correcting inconsistent entries ensuring that all symptom descriptions are standardized (Machen, 2023). Feature engineering is the process of creating new features or modifying existing features to improve model performance (Chiu et al., 2024). For our symptom-based health checker, we created binary features that indicate the presence or absence of a symptom. Moreover, normalization or scaling and data transformation are often applied as part of preprocessing. They involve scaling numerical features to have a certain distribution and converting data into a format that is suitable for a specific ML algorithm (Cofre-Martel et al., 2021). Feature selection involves identifying the most relevant features for predicting the target variable (Chiu et al., 2024), which in our case would be the health condition associated with the given symptoms. Recent studies have emphasized the importance of these steps. For instance, a study published in March 2024 highlighted the use of ML for the early prediction of cardiovascular disease (Machen, 2023). The researchers used advanced ML algorithms to discern predictive factors within electronic health data. This underscores the importance of data preprocessing and feature engineering in developing effective symptom-based health checkers (Cofre-Martel et al., 2021; Machen, 2023; Chiu et al., 2024).

### 2.3 Model selection and hyperparameters tuning

In this step, the constructed dataset is used to train the selected models. In our study, we have chosen those models: Decision Tree, Random Forest, Naive Bayes, Logistic Regression and K-Nearest Neighbors. Starting with Decision Trees, they are a popular choice in medical diagnosis due to their interpretability where each node in a decision tree represents a feature, such as a symptom or test result, and each branch represents a decision rule. This structure facilitates a clear visualization of the diagnostic process, which is essential for ensuring accuracy and effectiveness in a medical context (Yu et al., 2022; Faviez et al., 2024; Miao et al., 2024). Moreover, decision trees can handle both categorical and numerical data, accommodating a wide range of patient information. Random Forest is an ensemble of decision trees that further enhances the robustness of the model. Each tree in the random forest votes for a class and the class with the most votes becomes the model’s prediction. This approach is particularly suitable for medical diagnosis where datasets often have high dimensionality. Additionally, Random Forest provides a measure of feature importance identifying the most relevant symptoms or test results for a particular disease (Wongvibulsin et al., 2019; Chato and Regentova, 2023). On the other hand, Naive Bayes (Fauziyyah et al., 2020) classifiers offer computational efficiency and apply Bayes’ theorem with strong independence assumptions between the features. Despite their simplicity, Naive Bayes classifiers can be effective in predicting the probability of a disease given a set of symptoms or test results (Fauziyyah et al., 2020). Logistic Regression is another model that is commonly used in medical diagnosis able to predict the probability of either a binary or a multiple outcome. It can handle both categorical and continuous variables providing probabilities that can be interpreted as risk. It also captures the effect of different symptom combinations (Chen et al., 2023). Lastly, K-Nearest Neighbors (KNN) is a type of instance-based learning algorithm that classifies a new instance based on the majority class of its ‘k’ nearest instances in the feature space. KNN can be used to predict a patient’s disease status based on the disease status of similar patients and it is particularly useful when there is no prior knowledge about the distribution of the data (Tran and Ditto, 2020). Each of these models brings unique strengths to our health checker system. However, the performance of these models can depend heavily on the quality and characteristics of the data they are trained on. Therefore, we have conducted careful preprocessing and feature selection to overcome those issues. Model hyperparameter tuning (Chicco and Jurman, 2020; Shah et al., 2020) was a crucial step in our work to enhance the performances of our ML models. It involves adjusting the parameters that govern the training process itself rather than the model parameters learned from the data. We have performed this process using 10-fold cross-validation to ensure the generalization of the selected hyperparameters to unseen data. For Decision Tree, we have tuned the hyperparameters: maximum depth of the tree (max_depth), minimum number of samples required to split an internal node (min_samples_split), minimum number of samples required to be at a leaf node (min_samples_leaf). For Random Forest, we have tuned the hyperparameters: number of trees in the forest (n_estimators), maximum number of features to consider for splitting (max_features), and maximum depth of each tree (max_depth). Hyperparameter tuning has not improved Naive Bayes classifier’s accuracy due to the simplicity of the model. For Logistic Regression, we have tuned the hyperparameters: penalty hyperparameter and C hyperparameter that both work together in order to minimize the generalization error of the model and control overfitting, and solver hyperparameter determining the algorithm to use in optimization. For K-Nearest Neighbors, we have tuned the hyperparameter: number of neighbors to consider (n_neighbors), method used to calculate the distance (metric, e.g., Euclidean, Manhattan, etc.) and weight function used in prediction (weights, options include “uniform” or “distance”) The choice of the best hyperparameters can greatly influence the performance of the models and their efficient tuning is essential to achieve optimal results.

### 2.4 Model evaluation and benchmarking

#### 2.4.1 Model evaluation

After training a ML model, rigorous testing is essential to evaluate its performance using a variety of metrics. K-fold cross-validation is a fundamental method involving data partitioning into k subsets, training the model k times, each time using a different subset as the test set while the remaining subsets are used for training. This approach aids in evaluating the model’s robustness and generalization capabilities while mitigating the risk of overfitting (Zhang et al., 2022). The confusion matrix (Zhang et al., 2022; Prakash et al., 2024) is also a key tool for model evaluation which tabulates the true positives (TP), true negatives (TN), false positives (FP) and false negatives (FN) offering a comprehensive view of the model’s performance in classification tasks including multitask classification, which is the focus of our study. From this matrix, we derive several crucial metrics such as accuracy (Ceney et al., 2020) which measures the proportion of correctly classified instances out of the total instances. For imbalanced datasets, accuracy alone can be misleading and metrics like precision and recall become vital: precision indicates the ratio of correctly predicted positive observations to the total predicted positives, while recall (or sensitivity) measures the ratio of correctly predicted positive observations to all observations in the actual class. As harmonic mean of precision and recall, the F1 score provides a single metric that balances these two aspects especially useful for irregular class distribution. Receiver Operating Characteristic (ROC) curve and Precision-Recall (PR) curve are another sophisticated metrics plotting the true positive rate against the false positive rate and the precision against the recall at various threshold settings. Area under the Curve (AUC) is a comprehensive metric to evaluate classification models and to capture the model effectiveness in distinguishing between classes across all thresholds. The joint of all these metrics, often summarized in a classification report, offer a nuanced and comprehensive assessment of a model’s performance and effectively guide researchers in refining their models for better predictive accuracy and reliability (Liu et al., 2020, 2023). In our study, we combined all these metrics to evaluate the robustness of our health checker.

#### 2.4.2 Clinical vignette testing

For an online health checker, clinical vignette testing is a valuable tool to validate the ML models. The models are trained on a vast array of these clinical vignettes, learning to associate symptoms and patient history with potential diagnoses. This enables the online health checker to deliver more accurate and personalized health assessments. Moreover, clinical vignette testing serves as a benchmark to evaluate the performance of these ML models. By comparing the model’s predictions with expert-provided diagnoses for these vignettes, we can measure the model’s accuracy and we can prove the reliability of our health checker and its ability to handle a wide variety of real-world scenarios. In our work, we have used 10 clinical vignettes (Table 1) corresponding to the diseases being studied (Veloski et al., 2005; García-Carretero et al., 2020).

**Table 1:** Clinical vignettes.

Clinical vignette	Diagnosis
Vignette 1	Pertussis (Anh and Hong, 2019)
Vignette 2	Acute bronchitis (Marcio, 2024a)
Vignette 3	Pneumonia (Marcio, 2024b)
Vignette 4	Common Cold^a^
Vignette 5	Influenza (Heaney and Gallagher, 2020)
Vignette 6	Bronchiolitis (Mavridou and Laws, 2004)
Vignette 7	Gastroesophageal reflux disease (Atmaja et al., 2021)
Vignette 8	Acute sinusitis^b^
Vignette 9	Asthma (Ong et al., 2004)
Vignette 10	Chronic sinusitis (Sattar and Casillas, 2007)

^a^https://bestpractice.bmj.com/topics/en-gb/252/case-history [Accessed June 02, 2024].

^b^https://cpsa.ca/news/a-case-study-on-acute-rhinosinusitis-ars-do-no-harm [Accessed June 02, 2024].

#### 2.4.3 Health checker benchmarking

To further validate the performance of our models, it is essential to benchmark our health checker against existing similar platforms (Semigran et al., 2015). This comparative analysis helps us to identify areas of strength and potential improvement. It is a way to verify how our health checker meets or exceeds industry standards. We have cibled freely accessible online symptom checkers that are dedicated to human disease diagnosis. The criteria for our selection were as follows:

- The platforms are web-based applications,
- They had to be directly accessible to individuals without necessitating the creation of a user account,
- They should not be specialized toward single conditions such as diabetes, arthritis, pediatrics, etc.
- The platforms should be interactive, soliciting information from the user and providing responses, rather than merely offering condition information via an alphabetical list.

Thus, we targeted comprehensive, user-friendly platforms that could cater to a wide range of health concerns.

### 2.5 Solution architecture and model serving

**Figure 1:**
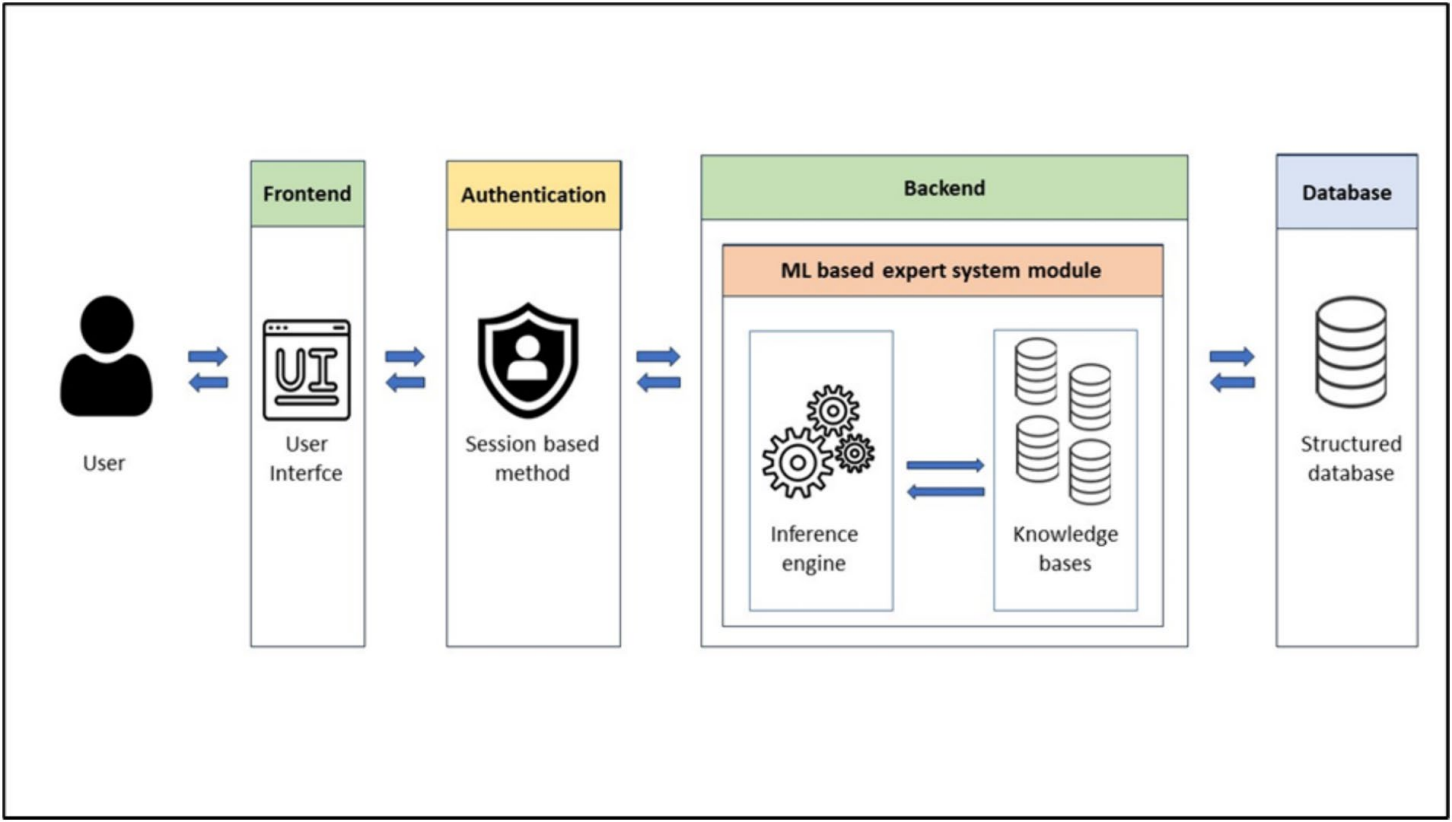
Solution architecture.

Our solution’s architecture (Figure 1) consists of four key components: FrontEnd, Authentication module, BackEnd, and the Database. The FrontEnd serves as the user interface allowing users to interact with the expert system engine and user requests are received and directed to the appropriate server-side endpoints. The BackEnd supplies the necessary endpoints for task execution and houses the ML module including the inference engine and the knowledge base. The knowledge base, designed to suit a ML-based expert system, contains a dataset of patient symptoms and diagnoses. To expedite the inference engine’s task, the global knowledge base is formed of many knowledge bases in a way that each knowledge base is specified in a particular chief complaint (cough, headache, dizziness, etc.). The inference engine uses this knowledge to generate system predictions. The Database stores user information, system predictions and doctors’ comments useful for user registration and sign-in processes. The Authentication module employs a session-based authentication method. This streamlined architecture ensures efficient and secure user interactions with our solution.

## 3 Results

### 3.1 Data collection and domain understanding

**Figure 2:**
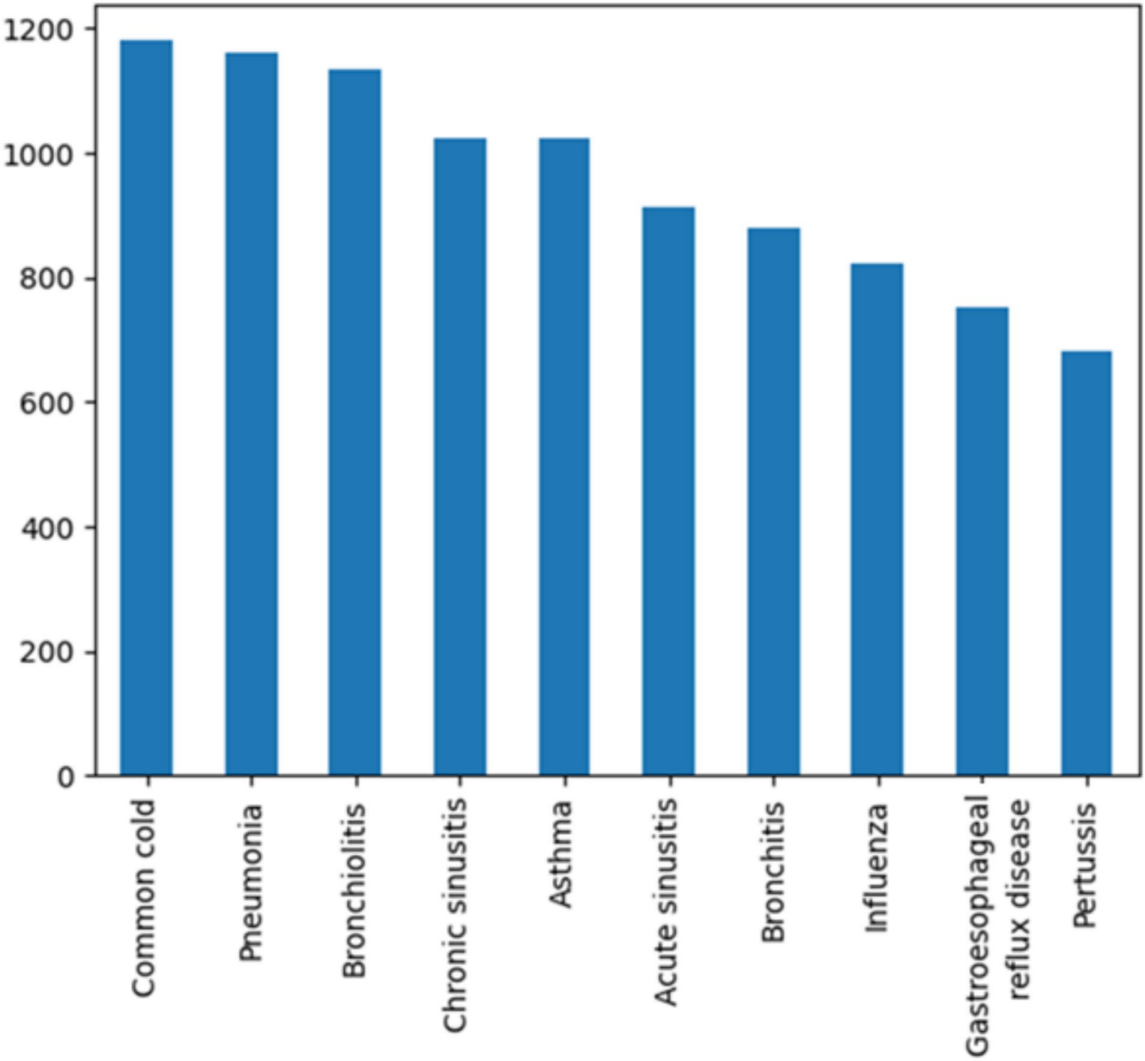
Constructed dataset.

Data collection aims to assemble a coherent dataset from one or more trustful sources like databases, mobile devices, records, files, etc. Our constructed dataset includes 10 diseases (Figure 2) with a relatively similar number of samples to avoid an imbalanced dataset.

We have allocated a significant portion of the samples (75%) to train our models ensuring efficient learning from a diverse range of diagnosis and we have also reserved 25% of samples for testing (Table 2) to evaluate model generalizability and robustness.

**Table 2:** Training and testing samples.

Condition	Number of samples for training	Number of samples for testing	Total
Influenza	609	212	821
Common cold	894	286	1,180
Chronic sinusitis	760	264	1,024
Gastroesophageal reflux disease	570	183	753
Pneumonia	852	310	1,162
Bronchitis	667	213	880
Acute sinusitis	683	228	911
Pertussis	520	162	682
Bronchiolitis	849	286	1,135
Asthma	775	249	1,024
Total	7,179	2,393	9,572

### 3.2 Data preprocessing and feature engineering

Data preprocessing and feature engineering are crucial steps in the development of a symptom-based health checker. In our study, we created binary features that indicate the presence or absence of a symptom. Feature selection identifies predictive factors which in our case include gender, age, risk factors and patient history associated with the given symptoms.

In our study, we focus on a common chief complaint: cough. We investigate the various causes that could lead to cough as a primary symptom. While there are multiple diseases that could explain cough, we are currently concentrating on a select few. The diseases that are included in our dataset are: Pneumonia, Asthma, Bronchitis, Common cold, Pertussis, Acute sinusitis, Gastroesophageal reflux disease, Chronic sinusitis and Bronchiolitis.

Taking into consideration the pros and cons of the shape of data in existing datasets addressed to symptom based medical diagnosis systems, the data in our dataset is designed to be clean from the start. It is organized in a way that the columns contain the possible symptoms (features) and the possible diagnosis (target) and that the rows represent samples of artificial patients. Each sample is a possible combination of some symptoms that may lead to a diagnosis. Our dataset is composed of 9,572 samples of artificial patients, 70 features describing the characteristics and the type of the cough in addition to the risk factors and the possible associated symptoms leading to the manifestation of a disease, and 10 possible diagnoses as targets. The distribution of samples in our current dataset after the execution of the “train_test_split (Dataset, test_size = 0.25, random_state = 0)” instruction is shown in Table 2.

### 3.3 Model selection and hyperparameters tuning

In our study, we selected the following models: Decision Tree, Random Forest, Naive Bayes, Logistic Regression, and K-Nearest Neighbors. The choice of optimal hyperparameters has significantly influence the performance of these models. After hyperparameter tuning, the selected models are:

Clf1: DecisionTreeClassifier (max_depth = 9, max_features = ‘sqrt’, min_samples_leaf = 3, min_samples_split = 8).
Clf2: RandomForestClassifier (max_depth = 8, max_features = ‘sqrt’, estimators = 1,000).
Clf3: MultinomialNB (force_alpha = True).
Clf4: LogisticRegression (C = 100, penalty = ‘l2’, solver = ‘newton-cg’).
Clf5: KNeighborsClassifier (metric = ‘euclidean’, n_neighbors = 1, weights = ‘uniform’).

Due to the large volume of model results, we have opted to present the results for Random Forest as a template, with the methodology being the same for all models. In Tables 3, 4, we present an illustration of the variation in Random Forest results achieved by employing different values for the max_depth hyperparameter, highlighting the corresponding performance enhancements.

**Table 3:** Random Forest results for different max_depth hyperparameter.

Max_depth	Accuracy on training set	Accuracy on testing set	f1_score (weighted avg)	f1_score (macro avg)
1	0.718	0.725	0.661	0.611
2	0.870	0.884	0.853	0.822
3	0.966	0.967	0.965	0.960
4	0.987	0.984	0.984	0.982
5	0.990	0.989	0.989	0.988
6	0.993	0.993	0.993	0.992
7	0.997	0.998	0.998	0.998
8	0.998	0.998	0.998	0.998
9	0.998	0.998	0.998	0.998
10	0.998	0.998	0.998	0.998

**Table 4:** 10-Fold Cross Validation for different max_depth hyperparameter.

Max_depth	10-Fold Cross Validation									
	Split1	Split2	Split3	Split4	Split5	Split6	Split7	Split8	Split9	Split 10
1	0.710	0.704	0.722	0.736	0.715	0.683	0.718	0.704	0.728	0.704
2	0.877	0.880	0.895	0.891	0.883	0.857	0.866	0.871	0.867	0.875
3	0.967	0.954	0.967	0.965	0.965	0.973	0.966	0.962	0.973	0.972
4	0.988	0.981	0.988	0.987	0.977	0.988	0.988	0.984	0.986	0.993
5	0.991	0.986	0.991	0.987	0.983	0.993	0.988	0.990	0.993	0.994
6	0.997	0.990	0.991	0.993	0.986	0.995	0.993	0.991	0.995	0.998
7	1	0.993	0.998	0.997	0.991	1	0.997	0.997	1	1
8	1	0.994	0.998	0.997	0.995	1	0.997	0.997	1	1
9	1	0.994	0.998	0.997	0.995	1	0.997	0.997	1	1
10	1	0.994	0.998	0.997	0.995	1	0.997	0.997	1	1

**Figure 3:**
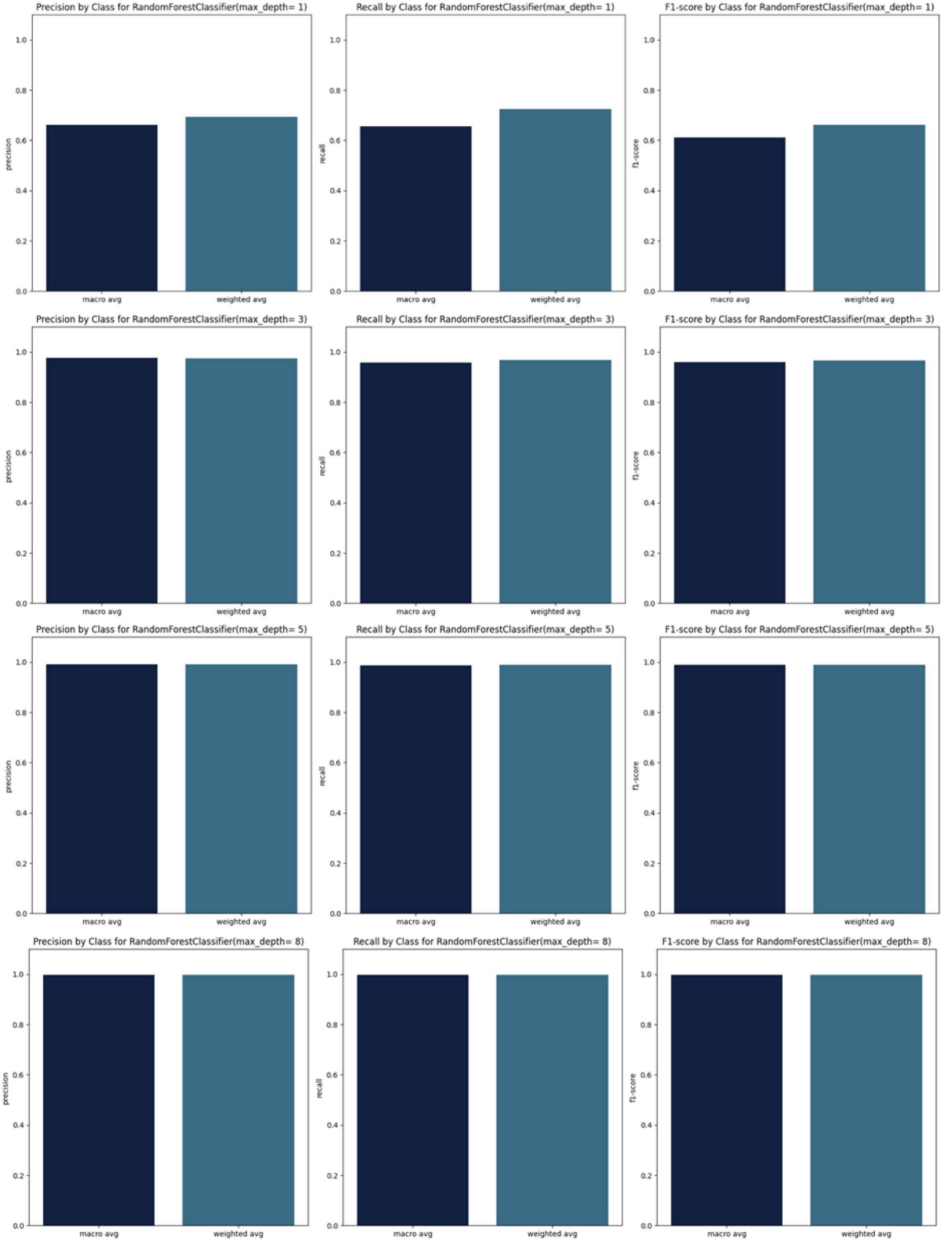
Precision, recall and F1_scores for RandomForest.

By incrementally increasing the max_depth parameter from 1 to 10 (Figure 3) in our Random Forest model, we observed a continuous improvement in precision, recall, and F1 scores. This suggests that as we allowed the decision trees to grow deeper, the model was better able to capture more complex relationships within the training data, resulting in improved ability to generalize and accurately predict classes in the test set. The ongoing enhancement in performance is a positive indicator of the effectiveness of our Random Forest model and its capacity to learn more discriminative patterns as the trees’ depth increases. However, this improvement may come with the risk of overfitting if the depth of the trees becomes too great. Therefore, careful evaluation is required to identify the optimal parameters that enhance the model’s performance while preserving its ability to generalize to new data.

**Figure 4:**
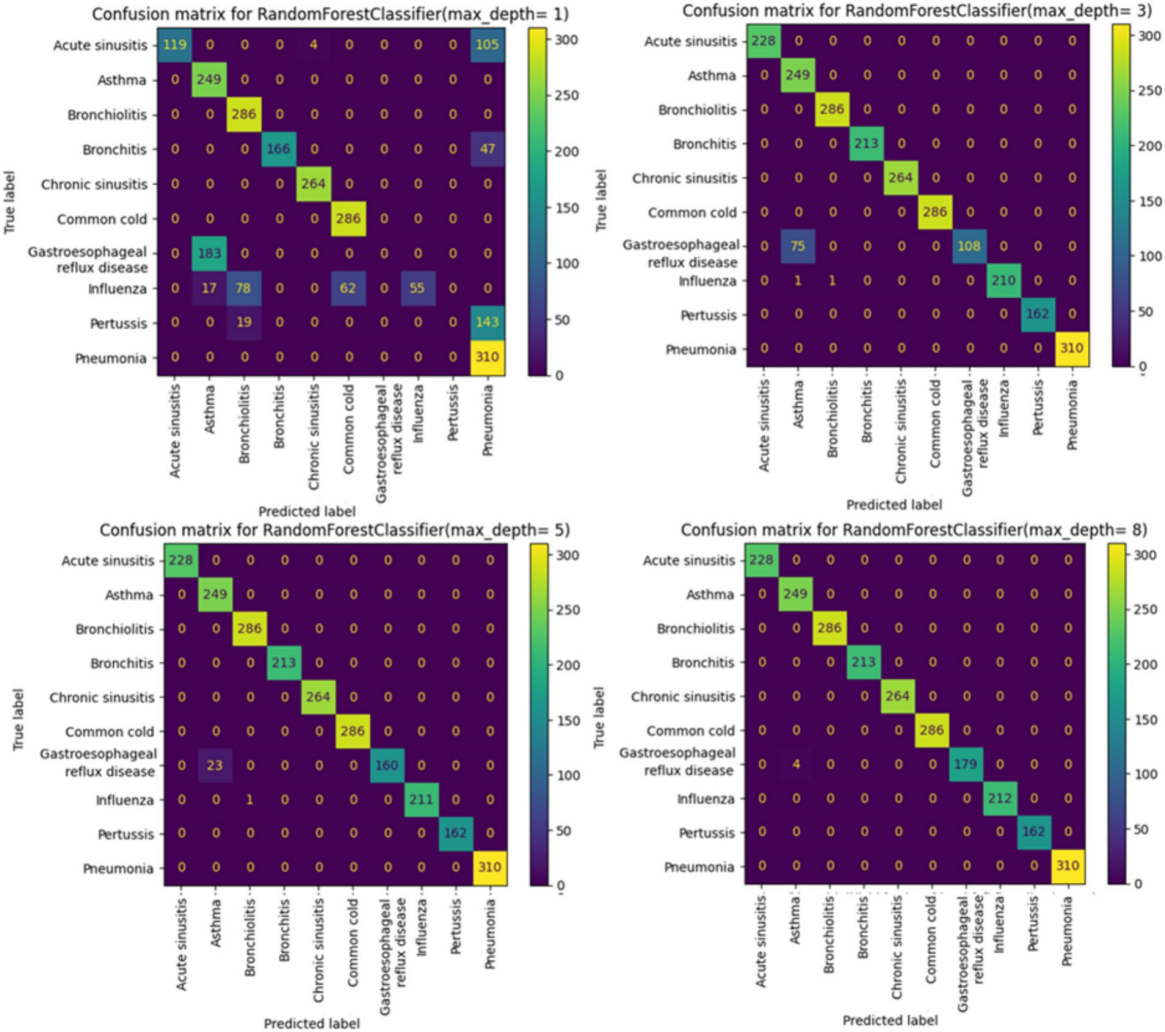
Confusion matrix for RandomForest.

Starting from max_depth = 8, the precision, recall, and F1 scores, as well as the confusion matrix, show strong performance (Figures 3,4).

**Figure 5:**
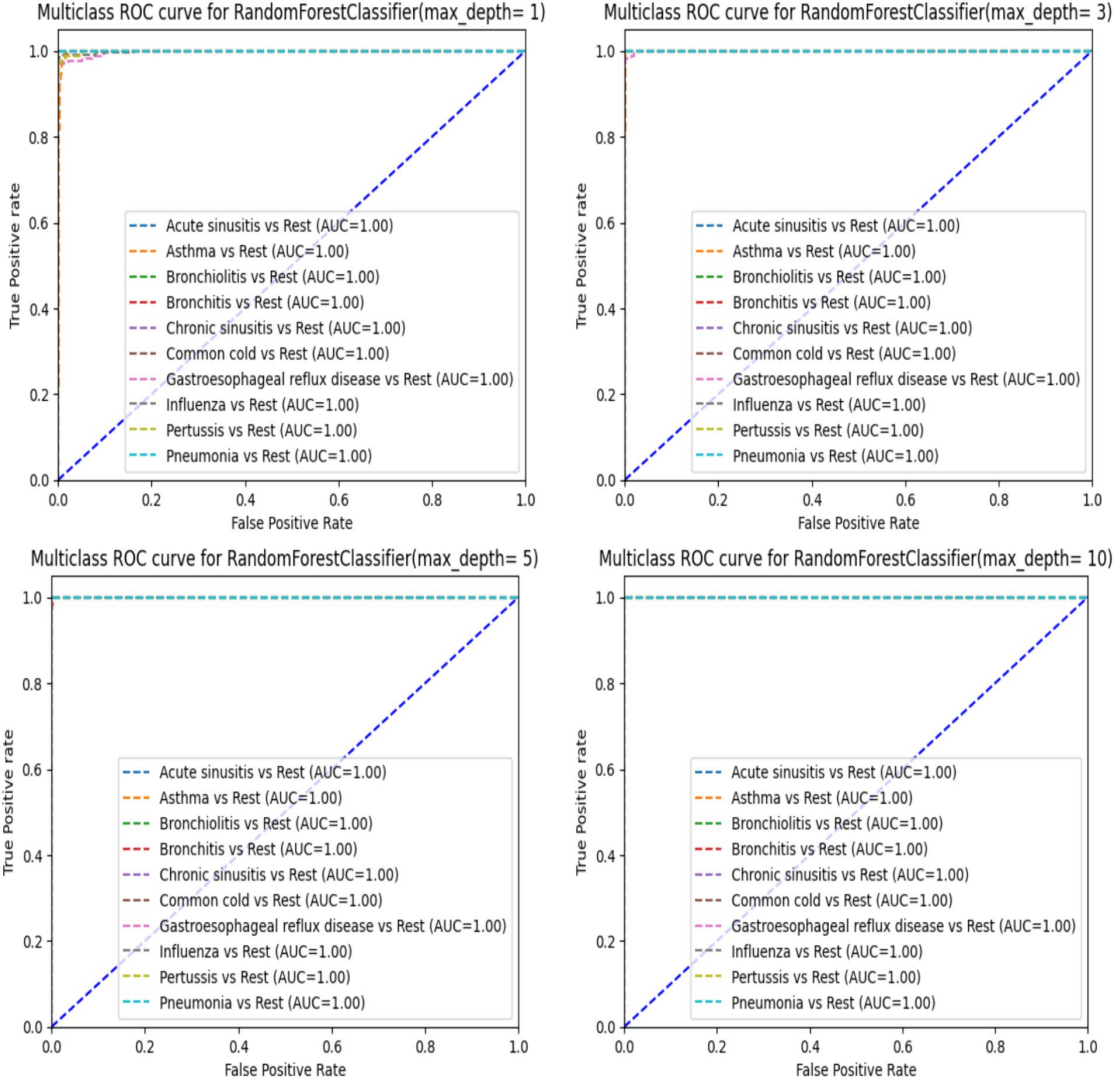
ROC-AUC curve for RandomForest.

When analyzing the performance of our Random Forest model using different max_depth values, we observed that the ROC-AUC curves (Figure 5) showed a significant improvement after max_depth = 1, with subsequent depths demonstrating strong performance. The initial underperformance at max_depth = 1 can be attributed to the model’s limited complexity. On the other hand, the precision-recall curves provide additional insights particularly for evaluating models on imbalanced datasets. Precision-recall curves are especially useful for highlighting performance differences in such scenarios, as they focus on the balance between precision and recall rather than just the overall accuracy.

**Figure 6:**
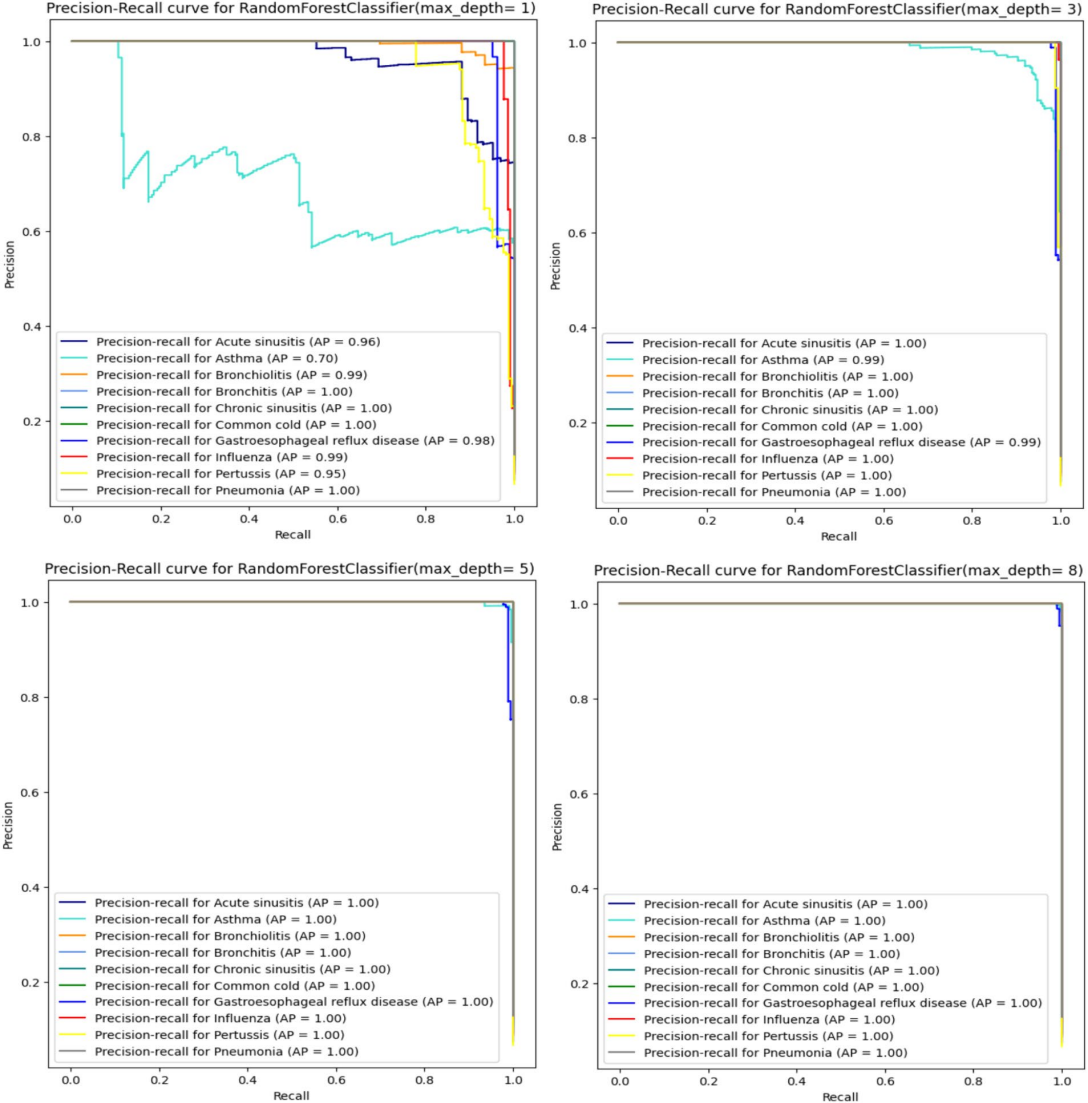
Precision-recall curve for RandomForest.

When using a Random Forest classifier with very low max_depth values less especially for value 1, the precision-recall curves indicate poor performance (Figure 6) particularly with one disease (Asthma) showing significantly worse results. This can be explained by underfitting. In fact, with low max_depth values, the Random Forest model is too simple to capture the complexity of the data and fails to learn important patterns in the data. Consequently, it cannot accurately distinguish between different classes leading to poor precision and recall. As max_depth increases, the model captures more complex patterns reducing false positives and improving the identification of true positives. This results in higher precision and recall, as reflected in the enhanced precision-recall curves (Figure 6). Starting from max_depth = 8, the precision-recall curve shows strong performance showing the model’s improved ability to handle imbalanced datasets and classes. In contrast, ROC curves, which plot true positive rate (sensitivity) against false positive rate (1-specificity) may not reveal the same degree of improvement for imbalanced classes. While both curves reflect model performance, precision-recall curves are more informative for imbalanced datasets, highlighting the enhanced precision and recall starting from max_depth = 8.

### 3.4 Model evaluation and benchmarking

#### 3.4.1 Model evaluation

The ML models have been evaluated through 10-fold cross validation, accuracy and f1_score metrics, and confusion matrices. Tables 5, 6 present the classification report for the selected models and show excellent performances (superior to 99%) for all the models except for the decision tree model that show lower scores for the accuracy and the F1 scores (93%).

**Table 5:** Classification report for the selected models.

Classifier	Accuracy on the training set	Accuracy on the testing set	f1_score (weighted avg)	f1_score (macro avg)
Clf1	0.937	0.933	0.932	0.934
Clf2	0.998	0.998	0.998	0.998
Clf3	0.997	0.996	0.996	0.996
Clf4	1	1	1	1
Clf5	1	0.999	0.999	0.999

**Table 6:** 10-Fold cross validation of the selected models.

Classifier	10-Fold cross validation									
	Split1	Split2	Split3	Split4	Split5	Split6	Split7	Split8	Split9	Split 10
Clf1	0.967	0.962	0.991	0.954	0.965	0.958	0.972	0.976	0.973	0.986
Clf2	1	0.994	0.998	0.997	0.995	1	0.997	0.997	1	1
Clf3	0.998	0.994	0.997	0.995	0.994	1	0.997	0.995	1	1
Clf4	1	1	1	1	1	1	1	1	1	1
Clf5	1	1	1	1	1	1	1	1	1	1

F1_score (weighted avg) calculates the F1 score for each class considering class imbalance and giving more weight to classes with more samples. F1_score (macro avg) calculates the unweighted average of F1 scores for all classes treating all classes equally regardless of their distribution.

All optimized models demonstrate excellent performance with 10-fold cross-validation with slight differences for all the models except for Clf1. Clf1 (DecisionTreeClassifier) has the lowest accuracy in both training set and testing set, and show varying accuracies across the 10 folds indicating it is less consistent and more susceptible to overfitting compared to the other models. Both Clf2 (RandomForestClassifier) and Clf3 (MultinomialNB) have very high accuracy showing high consistency. Clf4 (LogisticRegression) and Clf5 (KNeighborsClassifier) are the top-performing models across all splits.

#### 3.4.2 Clinical vignette testing

To evaluate our models for real-world use cases, we tested 10 clinical vignettes for each model. The results, highlighted in Table 7, show the rank and the presence of the correct diagnoses (provided by physicians) in the differential diagnosis list generated by our health checker (abbreviated as PRS in Table 7).

**Table 7:** Results of clinical vignette testing.

Classifier		Clf1		Clf2		Clf3		Clf4		Clf5	
		PRS	Rank	PRS	Rank	PRS	Rank	PRS	Rank	PRS	Rank
Vignette	V1	No	*	Yes	8	Yes	7	Yes	8	No	*
	V2	No	*	No	*	Yes	7	Yes	8	No	*
	V3	No	*	Yes	3	Yes	3	Yes	2	Yes	1
	V4	No	*	Yes	4	Yes	2	Yes	2	No	*
	V5	Yes	1	Yes	1	Yes	1	Yes	1	No	*
	V6	No	*	Yes	4	Yes	4	Yes	4	No	*
	V7	Yes	1	Yes	4	Yes	2	Yes	5	No	*
	V8	No	*	Yes	1	Yes	1	Yes	2	Yes	1
	V9	No	*	Yes	1	Yes	1	Yes	1	Yes	1
	V10	No	*	Yes	5	Yes	3	Yes	2	No	*

The results highlight a varying performances among the five classifiers (Clf1 to Clf5). Both Clf3 and Clf4 excelled and correctly diagnosed 10 out of 10 vignettes with consistently high rankings making them highly suitable and reliable for real-world diagnostic applications. Clf2 also performed well and correctly diagnosed 9 out of 10 vignettes with varied rankings but it remains a strong contender for clinical use. Nevertheless, Clf1 is unreliable for real-world applications and demonstrated poor performance with only 2 correctly diagnoses out of 10 vignettes. On the other hand, Clf5 correctly diagnosed 3 out of 10 vignettes and often ranked correct diagnoses at the top but still falls short of the accuracy required for clinical reliability.

#### 3.4.3 Health checker benchmarking

The selected symptom checkers are listed in Table 8. It was observed that all the mentioned symptom checkers follow three main steps:

- Step 1: Entering primary symptoms to initiate the evaluation process.
- Step 2: Responding to related questions about these symptoms to gather additional information.
- Step 3: Presenting predictions for both diagnosis and triage (Berry et al., 2019, 2022; Shen et al., 2019) based on the entered information.

**Table 8:** List of some web-based symptom checkers.

Symptom checker	URL
Ubie health	https://ubiehealth.com
Symptomate	https://symptomate.com
Docus	https://docus.ai/symptom-checker
Isabel	https://symptomchecker.isabelhealthcare.com
WebMD	https://symptoms.webmd.com

It was noted that there is no standardized method for symptom checkers to interact with users, and there are various approaches to displaying the system’s results for diagnosis and triage.

In the benchmarking of symptom checkers presented in Table 9, we observe varying performance levels between our top classifiers (Clf3 and Clf4) and commercial symptom checkers. While Ubie Health displayed moderate reliability with mixed results across vignettes, Symptomate exhibited consistent performance ranking well in most cases. Docus demonstrated good overall performance, consistently achieving high rankings in several vignettes. On the other hand, Isabel showed mixed performance with some high rankings but lower rankings in other cases. WebMD’s results were inconsistent, with varying rankings across different vignettes. In comparison, Clf3 (MultinomialNB) and Clf4 (LogisticRegression) consistently performed well achieving high rankings across multiple vignettes. Their reliable performance suggests their potential for real-world diagnostic applications outperforming the tested commercial symptom checkers in terms of consistency and accuracy.

**Table 9:** Benchmarking results: comparative analysis with our health checker (Clf3 & Clf4).

Classifier		Ubie		Symptomate		Docus		Isabel		WebMD		Clf3		Clf4	
		PRS	Rank	PRS	Rank	PRS	Rank	PRS	Rank	PRS	Rank	PRS	Rank	PRS	Rank
Vignette	V1	Yes	7	Yes	4	No	*	No	*	Yes	18	Yes	7	Yes	8
	V2	Yes	3	Yes	4	Yes	1	Yes	1	Yes	2	Yes	7	Yes	8
	V3	Yes	4	Yes	1	Yes	1	Yes	9	Yes	1	Yes	3	Yes	2
	V4	Yes	2	Yes	1	Yes	1	Yes	2	Yes	2	Yes	2	Yes	2
	V5	No	*	Yes	1	Yes	1	Yes	2	Yes	1	Yes	1	Yes	1
	V6	No	*	No	*	No	*	Yes	11	No	*	Yes	4	Yes	4
	V7	Yes	2	Yes	8	Yes	2	Yes	9	Yes	1	Yes	2	Yes	5
	V8	Yes	1	Yes	2	Yes	1	Yes	1	Yes	3	Yes	1	Yes	2
	V9	Yes	1	Yes	1	Yes	1	Yes	1	Yes	2	Yes	1	Yes	1
	V10	Yes	1	Yes	1	Yes	1	Yes	4	Yes	2	Yes	3	Yes	2

### 3.5 Solution architecture and model serving

**Figure 7:**
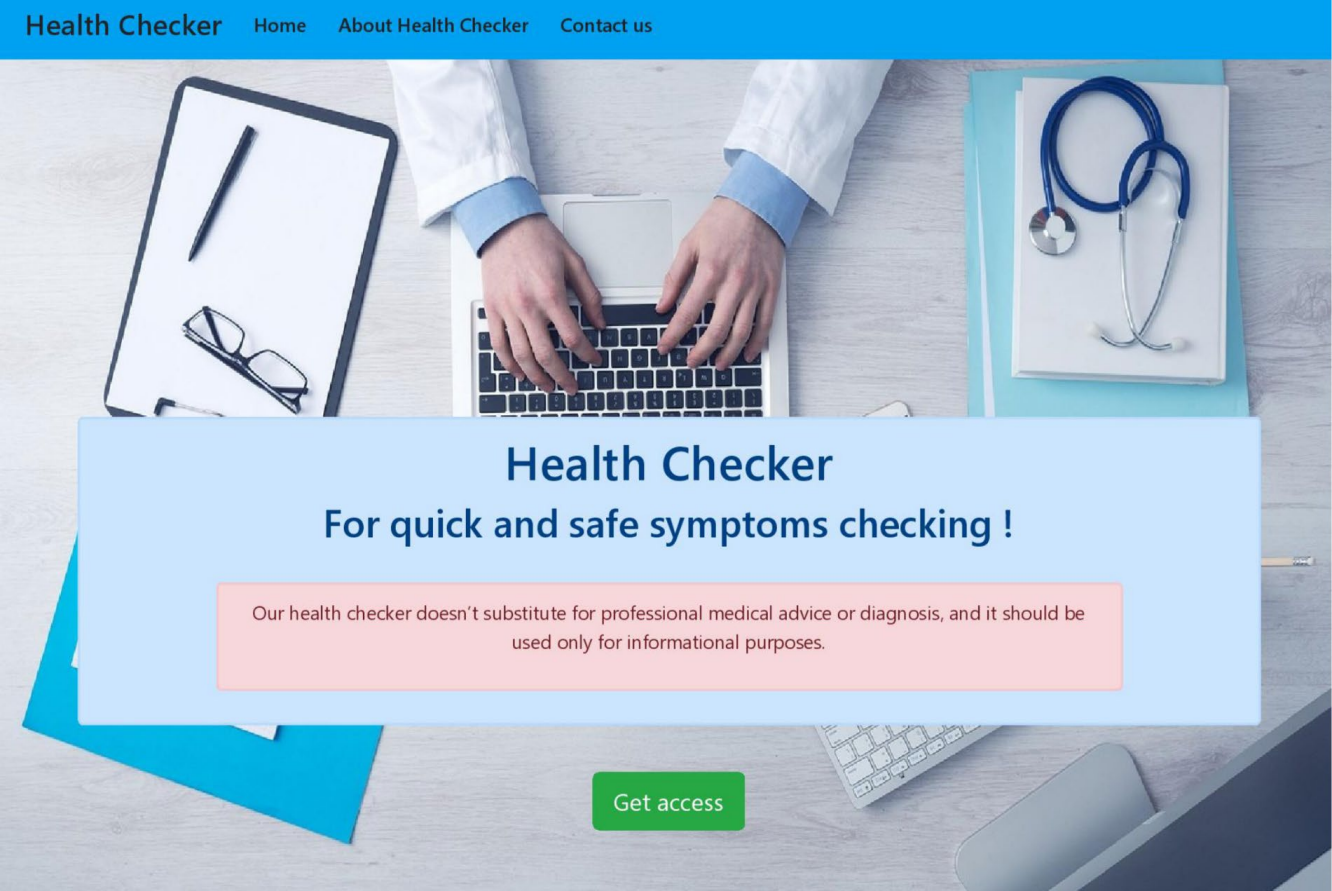
Health checker home page.

Figure 7 shows the home page of our use-friendly health checker.

Some of the tested symptom checkers (Table 6) were clinically validated and renowned for their accuracy in predicting the causes of patients’ experienced symptoms like Isabel Healthcare and our health checker. The majority of these applications were the result of the cooperation between physicians and data scientists. For the safety of users, on each symptom checker’s website, it is mentioned that the symptom checker does not substitute for professional medical advice or diagnosis, and it should be used only for informational and educational purposes.

## 4 Discussion

In this study, we developed and evaluated a symptom-based health checker designed to diagnose 10 respiratory and digestive systems related diseases based on symptoms, particularly focusing on cough as a chief complaint. Our comprehensive constructed dataset, careful model selection and rigorous evaluation process allowed us to achieve significant insights and promising results for real-world diagnostic applications proved through clinical testing and benchmarking with existing commercial health checkers.

In addition to accuracy and F1 scores, we employed confusion matrices and ROC-AUC curves to further evaluate our models. These metrics provided deeper insights into the performance of each classifier. Confusion matrices helped us understand the distribution of true positives, true negatives, false positives, and false negatives, revealing the model’s strengths and weaknesses in classifying each condition. The evaluation of our Random Forest model using ROC-AUC curves revealed a significant improvement in performance beyond a max_depth of 1, with subsequent depths maintaining strong results. This indicates the model’s enhanced ability to distinguish between classes as its complexity increases. However, precision-recall curves provided a deeper insight, especially for lower max_depth values (1, 2, and 3), where they highlighted poor performance in identifying true positives for certain diseases. The divergence between these metrics highlights the importance of using both ROC-AUC and precision-recall curves, especially in the context of imbalanced datasets. While ROC-AUC curves offer a broad measure of model discrimination, precision-recall curves are crucial for understanding the trade-off between precision and recall, ensuring more reliable and sensitive diagnostic performance.

During clinical testing, the evaluation of our ML models demonstrated that results highlight a varying performances among the five classifiers (Clf1 to Clf5). The difference in performance among the models can be attributed to several factors including the characteristics of each ML model, the number of clinical vignettes and the inherent complexity of the data. Clf1, a Decision Tree classifier, tends to overfit the training data which limits its ability to generalize explaining its poor performance with only 2 correct diagnoses out of 10. The limited number of clinical vignettes might intensify this issue due to lack of enough diverse examples.

Clf2, a Random Forest classifier, benefits from ensemble learning enhancing robustness and generalization and resulting in 9 correct diagnoses but with some variability in ranking. This variability might be due to the way different trees in the forest prioritize features leading to inconsistent rankings across different vignettes.

Clf3, a Multinomial Naive Bayes classifier, performed exceptionally well with 10 correct diagnoses. Its probabilistic nature and simplicity make it effective for handling class imbalances and providing reliable classifications. It also tends to perform well even with a smaller number of samples which could explain its high accuracy.

Clf4, a Logistic Regression model, also diagnosed 10 out of 10 vignettes correctly. Its ability to model linear relationships and manage complex interactions between features contributes to its high performance. Logistic Regression is generally robust to smaller datasets maintaining accuracy even with fewer vignettes.

Clf5, a K-Nearest Neighbors classifier, showed moderate performance with 4 correct diagnoses. While effective in some cases, KNN can struggle with more complex data distributions and lacks the consistency needed for clinical reliability. KNN’s performance heavily depends on the local distribution of data points and with a limited number of vignettes, it might not have enough neighbors to make accurate predictions.

Overall, the high performance of Clf3 and Clf4 makes them suitable for real-world diagnostic applications while Clf1 and Clf5 fall short of the required accuracy. Factors such as model complexity, susceptibility to overfitting, handling of class imbalances, and the limited number of vignettes all play a role in these performance differences.

Our benchmarking against commercial symptom checkers revealed that our optimized Naive Bayes and Logistic Regression models outperformed existing solutions such as Ubie Health, Symptomate, Docus, Isabel, and WebMD. While some commercial checkers like Symptomate and Docus demonstrated good performance with high rankings in several vignettes, others like Isabel and WebMD showed inconsistent results and highlighting the variability in commercial solutions’ diagnostic accuracy and reliability.

The consistently high performance of our health checker indicates that it could serve as a reliable alternative or complement to existing commercial symptom checkers, potentially providing users with more accurate and dependable diagnoses. The comparative Table 10 evaluates different health checkers (Ubie, Symptomate (Infermedica), Docus, Isabel and WebMD) and our health checker based on diagnostic performance. Performance is measured by the percentage of cases where the correct diagnosis (Dg) is ranked in the top 3, top 5, or included in the list of proposed diagnoses. The data come from various studies and are compared among the health checkers to assess their accuracy (Berry et al., 2019, 2022; Shen et al., 2019). The missing data are due to gaps in the available research.

**Table 10:** Comparative analysis of health checkers’ across studies.

	Ubie			Symptomate (Infermedica)			Docus			Isabel			WebMD			Our health checker		
	Dg in top 3	Dg in top 5	Dg in the list	Dg in top 3	Dg in top 5	Dg in the list	Dg in top 3	Dg in top 5	Dg in the list	Dg in top 3	Dg in top 5	Dg in the list	Dg in top 3	Dg in top 5	Dg in the list	Dg in top 3	Dg in top 5	Dg in the list
Aissaoui Ferhi et al. (2024)	60	70	80	60	80	90	80	80	80	50	60	90	80	80	90	70	80	100
Ceney et al. (2020)	–	–	–	–	>70	–	–	–	–	–	>70	–	–	>60	–	–	–	–
Semigran et al. (2015)	–	–	–	34	–	34	–	–	–	69	–	84	51	–	62	–	–	–
Berry et al. (2019)	–	–	–	7.7	–	8.9	–	–	–	16.1	–	39.3	17.3	–	36.3	–	–	–
Shen et al. (2019)	–	–	–	–	–	–	–	–	–	–	–	–	40	–	57	–	–	–
Berry et al. (2022)	–	–	–	33.3	-	33.3	–	–	–	33.3	–	80	66.7	–	93.3	–	–	–

Our health checker exhibits notable performance across different diagnostic accuracy metrics:

- Top 3 diagnoses: our health checker achieves a strong performance with 70% of correct diagnoses appearing in the top 3 positions. This indicates that the correct diagnosis is among the top three suggestions in 70% of cases, demonstrating a significant level of accuracy in providing the most likely diagnoses early in the list.
- Top 5 diagnoses: the performance improves further with 80% of correct diagnoses appearing in the top 5 positions. This shows that our health checker is effective in including the correct diagnosis within the top five suggestions in 80% of cases, enhancing the likelihood of users receiving relevant diagnostic options.
- In the list: the performance of our health checker is exceptional, with 100% of correct diagnoses appearing somewhere in the complete list of proposed diagnoses. This means that every correct diagnosis is included in the overall list of suggestions, ensuring that users always have access to the correct diagnosis, even if it’s not among the top suggestions.

To further enhance our health checker, future research should focus on the following:

- Diverse real-World data integration by incorporating real-world patient data from diverse sources such as electronic health records and mobile health applications can improve the model’s accuracy and generalization.
- Expansion of disease overage by extending the range of diagnosable conditions to include more diseases and symptoms can broaden the utility of the health checker.
- Longitudinal studies by conducting longitudinal studies to track the health checker’s performance over time and across diverse populations will provide valuable insights into its effectiveness and areas for improvement.

## 5 Conclusion

In conclusion, our symptom-based health checker has made several significant contributions to the field. Firstly, it has demonstrated exceptional precision and robustness, particularly through the effective application of Naive Bayes and Logistic Regression models. We have validated the reliability of these models using confusion matrices and ROC-AUC curves, highlighting their effectiveness in diagnostic applications. This study lays a solid foundation for developing diagnostic tools that are not only accurate but also user-friendly. Our work contributes to the body of knowledge by advancing methodologies in health checker development, particularly through rigorous validation techniques and the integration of high-quality datasets. Future research will build on this foundation by incorporating diverse real-world data, expanding disease coverage, and exploring advanced modeling techniques, thereby enhancing the health checker’s utility and impact in both clinical and consumer health contexts.

## Data availability statement

The raw data supporting the conclusions of this article will be made available by the authors, without undue reservation.

## Author contributions

LA: Conceptualization, Data curation, Formal analysis, Funding acquisition, Investigation, Methodology, Project administration, Resources, Software, Supervision, Validation, Visualization, Writing – original draft, Writing – review & editing. MB: Conceptualization, Data curation, Formal analysis, Funding acquisition, Investigation, Methodology, Project administration, Resources, Software, Supervision, Validation, Visualization, Writing – original draft, Writing – review & editing. FC: Methodology, Supervision & Validation, Writing – review & editing. RB: Methodology, Supervision & Validation, Writing – review & editing.

## Footnotes

^1^https://www.kaggle.com/datasets/kaushil268/disease-prediction-using-machine-learning [Accessed 01 June 2024].

^2^https://data.mendeley.com/datasets/dv5z3v2xyd/1 [Accessed June 02, 2024].

^3^https://www.vidal.fr [Accessed June 02, 2024].

^4^https://www.mayoclinic.org [Accessed June 02, 2024].
